# How urbanisation alters the intensity of the urban heat island in a tropical African city

**DOI:** 10.1371/journal.pone.0254371

**Published:** 2021-07-13

**Authors:** Xueqin Li, Lindsay C. Stringer, Sarah Chapman, Martin Dallimer

**Affiliations:** 1 Department of Environment and Geography, University of York, York, United Kingdom; 2 Institute for Climate and Atmospheric Sciences (ICAS), School of Earth and Environment, University of Leeds, Leeds, United Kingdom; 3 Sustainability Research Institute, School of Earth and Environment, University of Leeds, Leeds, United Kingdom; Northeastern University (Shenyang China), CHINA

## Abstract

Due to the combined effects of urban growth and climate change, rapid urbanisation is particularly challenging in African cities. Areas that will house a large proportion of the urban population in the future coincide with where natural hazards are expected to occur, and where hazard risk management institutions, knowledge, and capacity are often lacking. One of the challenges posed by rapid urbanisation is the Urban Heat Island (UHI) effect, whereby urban areas are warmer than the surrounding rural areas. This study investigates urbanisation patterns and alterations in surface UHI (SUHI) intensity for the Kampala urban cluster, Uganda. Analyses show that between 1995 and 2017, Kampala underwent extensive changes to its urban built-up area. From the centre of the city to adjoining non-built up areas in all directions, the urban land cover increased from 12,133 ha in 1995 to 25,389 ha in 2016. The area of SUHI intensity in Kampala expanded significantly over the 15-year period of study, expanding from 22,910 ha in 2003 to 27,900 ha in 2016, while the annual daytime SUHI of 2.2°C in 2003 had decreased to 1.9°C by 2017. Although SUHI intensity decreased in some parts of the city, elsewhere it increased, suggesting that urbanisation does not always lead to a deterioration of environmental conditions. We postulate that urban development may therefore not necessarily create an undesirable impact on local climate if it is properly managed. Rapidly growing cities in Africa and elsewhere should ensure that the dynamics of their development are directed towards mitigating potentially harmful environmental impacts, such as UHI effect through careful planning that considers both bluespaces and greenspaces.

## Introduction

One of the major phenomena associated with the dual challenges of climate change and urbanisation is the urban heat island (UHI) effect, which is when the temperature in urban areas is higher than surrounding suburban and rural areas [1, 2]. The differences in the urban and rural areas’ energy balance results in the UHI effect. Urbanisation processes inevitably increase impervious surfaces, encroache on green and bluespaces, and alter albedo and geometry compared to rural surfaces [3, 4]. The UHI effect not only alters the environment in urban areas including net primary production, biodiversity, water and air quality [5, 6], but also affects human health and well-being, increasing the risk of heat-related morbidity and mortality [7, 8]. Typically, urban heat problems will be exacerbated by the combined impact of heat waves and UHI, when taking future climate projection into consideration [9].

According to the methodological approaches to investigate temperature differences between urban and rural areas, UHI can be described as the subsurface heat island, air heat island or surface heat island [10–12]. The subsurface heat island refers to the coupling between surface air temperatures and the ground surface temperature [13]. The air heat island represents UHI effects in the urban canopy layer or the urban boundary layer [14]. The canopy UHI measurement is mainly based on situ sensors mounted from ground to roof level (aeronautical meteorological stations or vehicle-mounted sensors) [10, 11]. The boundary layer UHI measurement relies on special platforms above the roof level (tall towers, radiosondes, tethered balloon flights and aircraft) [12]. Developing and installing such measurement stations is expensive and time-consuming. This means that they are only used in a few large cities around the world. Due to this limiting factor, insufficient data on atmospheric UHIs usually fail to provide adequate spatial details for land use strategies and climate change management in urban areas of most developing countries [15]. The surface heat island is also known as surface urban heat island (SUHI), and is measured from land surface temperature (LST), which is primarily observed by satellite thermal remote sensing data [16]. Advancements in remote sensing and spatial information sciences provide researchers with consistent and repeatable observation data to analyse the urban thermal environment at various spatial and temporal scales [17, 18].

East Africa has witnessed rapid urbanisation over the past few decades, which is likely to continue into the future [19]. Cities in this region will undergo enormous change in urban infrastructure and population, leading to increases in UHI intensity, with implications for heat stress, public health, and cooling energy consumption [20], particularly under projected climate changes. Various studies have documented the UHI effect in selected cities in East Africa (e.g. for Dar es Salaam [21]; Nairobi [22] and Addis Ababa [23, 24]) but knowledge gaps remain. Two crucial environmental challenges rapidly expanding cities are facing relate to the need to prevent flooding after heavy rain, and to reduce excessive heat due to the UHI effect [25, 26]. There is a complex relationship between these two phenomena, as rainfall increases with rising air temperature [26]. Indeed, the UHI effect and higher flood risk are different aspects of urban development issues, and need to be addressed together. UHI effects are often associated with, or even drive, more intense rainfall events over cities [27–30], and could therefore result in the greater likelihood of flooding. While studies in the literature focus on other locations, quantification and analysis of the magnitude and spatial pattern of UHI effects in case study cities in East Africa are less well studied. Such analyses can not only help in further comprehending the characteristics and driving forces of the SUHI effect, which is already reasonably well understood [8], but will also be crucial for seizing opportunities for land-based adaptation and to develop optimal climate mitigation strategies in the region. This paper adds a valuable East African analysis to the growing body of literature, focusing on the Ugandan capital city, Kampala, investigating the relationship between urbanisation and SUHI intensity change. The main objectives of the study are to: a) evaluate the urban growth pattern of Kampala, b) calculate the variations of annual SUHI intensity of Kampala, and c) explore how urban expansion results in SUHI intensity change and how the transformation of urban areas influences the SUHI.

## Method

### Study area and data sources

As one of the most densely populated areas in East Africa, the Great Lake Victoria Region (GLR) has over 30 million residents [31, 32]. Over the past decade, this region has experienced radical urbanisation, with large influxes of people to urban areas [33]. All countries in GLR are projected to experience increases in the duration, frequency and intensity of extreme heat events, with parts of Kenya, Uganda, and the Democratic Republic of Congo expected to be the locations with the highest growth [34]. Because of the complex and dynamic land-lake-atmosphere interactions, several studies highlight the importance of climate relevant research over the GLR [35, 36]. Since climate change is expected to stimulate change to land and lake breezes and to have significant impacts on local communities [37–39], associated possible extreme weather and climate events [36, 40] may cause severe socio-economic impacts on vulnerable groups [41]. To support the development of proper mitigation and adaptation strategies, it is imperative to understand the impacts of rapid urbanisation in this part of Africa.

**Fig 1:**
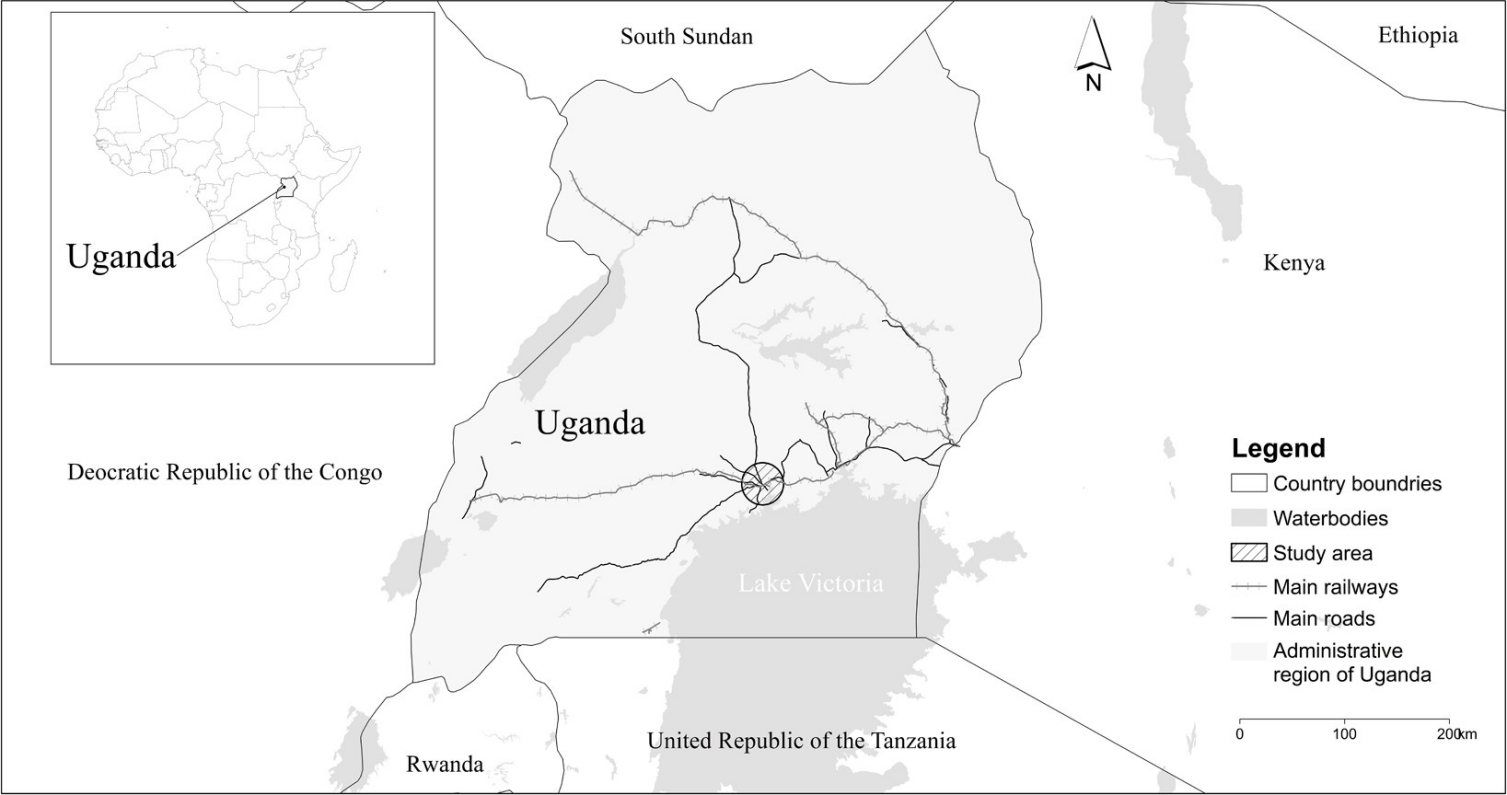
The location of Kampala in Uganda and Africa.

Kampala, the capital and main commercial centre of Uganda, is one of the most populated and fastest growing cities in East Africa (Fig 1). The city’s population was nearly 1.75 million in 2015 [54], and has been estimated to be growing at an average annual rate of 1.97% between 2015 and 2021 [42]. Characterized by the presence of uncontrolled informal settlements [43], Kampala is located on the northern shore of Lake Victoria at a mean altitude of 1203 m above sea level. The urban topography is characterized by rounded hills and wetlands, while the urban structure is shaped by the socio-economic stratification of wealth: richer neighbourhoods are located typically at the top of the hills, and poorer areas are at lower altitudes, closer to wetlands [39, 44]. Residential density has been reported to increasingly be concentrated on the hillslopes, with the wetlands gradually being encroached and filling with informal settlements, in spite of wetland protection laws [39, 45]. Flooding is the major climate hazard experienced by Kampala [46, 47]. However, Kampala is also defined as one of the region’s most vulnerable cities when it comes to heat stress [48]. Urbanisation and climate change could cause heat stress [34, 49] and flood risks [25, 50, 51] to increase. These two challenges could both be magnified by the SUHI effect [52, 53].

In this research, three kinds of data were linked within the analysis. Firstly, we used Landsat imagery through Google Earth Engine (GEE) to quantify the extent of the impervious surfaces associated within the city. The urbanized extent of Kampala is already larger than the administrative boundary of the city [39]. We therefore based our research on the built-up area within a 20 km-radius from the city centre [54, 55]. Then, we used MODIS average 8-Day (MOD11A2.006) within a zone 100 km^2^ from city centre through GEE to detect the annual daytime LST change in Kampala. Finally, we used the Global Surface UHI Explorer within GEE to retrieve annual daytime SUHI intensity, during this step, the “urban cluster” concept was used to compare the urban-rural LST differences [56, 57]. Data sources and processing steps, and the overall methodology are summarised in next section and Table 1.

**Table 1:** Data sources used to examine historical patterns of urbanisation in Kampala, Uganda.

	Data Layer	Spatial Resolution (m)	Date	Scale
**Built-up**	Landsat 8 Surface Reflectance (SR)	30	2017	20 km-radius subset from the city centre
	Landsat 5 SR	30	1995, 2010	
	MODIS Land Cover Type	500	2010	
**LST**	MOD11A2.006	1000	2003, 2017	100 km^2^ square from city centre
**SUHI**	Global Surface UHI Explorer	1000	2003, 2017	Kampala urban cluster

### Historical patterns of urbanisation

To understand how SUHI intensity and location altered with increased urbanisation, we examined historical patterns of urbanisation in Kampala by quantifying the extent of the impervious surfaces associated with the city. The extent of impervious surfaces can be measured unambiguously by remote sensing data, and therefore can be used in a consistent manner to compare urban areas over time and space [58]. We used Landsat imagery available through Google Earth Engine (GEE) to assess the changes in urbanisation across the Kampala urban cluster. Landsat data were processed using the cloud-computing technology in the GEE platform (https://code.earthengine.google.com/). Kampala’s economic zone extends to about 20 km from the city centre and 40 km along the rapidly developing peninsula up to Entebbe Airport [59]. We therefore calculated the built-up within a 20 km radius subset from the city centre [54, 55]. The islands in the Lake that fell with the urban cluster were also included.

Landsat imagery scenes were selected based on availability of high-resolution aerial imagery for Kampala. Many Landsat images inevitably suffer from cloud contamination in the tropics [60], which reduces the accuracy in land cover classification and land cover change [61]. To select Landsat scenes that are clear from cloud and cloud shadow, the Fmask (Function of mask) algorithm was applied by selecting the best cloud-free pixel. Due to cloud contamination, no images could be found that were suitable for extracting urban land cover in the years 2003 and 2017. Landsat imagery scenes were used from 1995, 2010 and 2016. To enhance the classification of results and ensure an objective identification of the Landsat imagery data, we used MODIS Land Cover Type 2010 in this study. Since the reference dataset is an important consideration in terms of the accuracy of remotely sensed data. When analysing the imagery, the random forest classifier with 10 trees was used to downscale MODIS data to Landsat resolution and generating two random samples (training and validation) from the MODIS data. The training sample is used to train the classifier [62]. From the validation sample, we assessed the classifier performance by Confusion Matrix [63], and set the limits of the training overall accuracy as 90% and the validation overall accuracy as 80%. Training overall accuracy ranged from 0.906 in 1995 to 0.967 in 2016; validation overall accuracy ranged from 0.814 in 2016 to 0.906 in 1995, indicating that overall accuracy is good.

After obtaining the urban built-up classification result, we estimated the intensity of urban built-up area coverage across the Kampala urban cluster using the kernel density (KD) function of ArcGIS Spatial Analyst. Generally, the KD tool estimates the density of point features around each output raster cell [64]. This measures Euclidean distances by calculating a smoothly continuous curved surface to fit the frequency distribution [65–67]. In the visualizations provided by KD estimation, the peak of the surface value is the location of the point representing the presence of clusters or ‘hot spots’ in the distribution of events [64, 68]. The surface values diminishing with increasing distance from the point represent events that occur much less frequently in the area [64, 68]. KD has often been used for the purpose of point-pattern analyses, such as population analysis [69], crime hot spots analysis [65, 70] and wildlife management [71]. It is consequently becoming more common to use a KD approach to study ecological networks and landscape changes [68, 72–74]. The results of KD estimation directly reflect the spatial patterns of the mutation in the built-up area [75]. ArcGIS uses the KD function calculation (Eq (1)), originally developed by Silverman in 1986 [64, 76]:

$$f_{n}(x)=\frac{1}{nh}\sum_{i=1}^{n}k\left(\frac{x-xi}{h}\right)$$ (1)

Where *f_n_*(*x*) is the estimated value of probability density of point *x, k*() is the KD function, *h* (*h* > 0) is the bandwidth of the estimation model, which is the radius of the analysis neighborhood; and (*x−xi*) is the distance from the estimation point to the sample *xi* [75]. In KD estimation, the bandwidth *h* plays an important role on the computed outcome. The dot density in the space becomes smoother accompany with the *h*, which may reduce the density structure. Conversely, the estimation point distributes uneven when *h* decreases [68]. In this study, we used the 30m bandwidth.

### Estimation of land surface temperatures and the SUHI effect

We used MODIS Collection-6 MODIS /Terra Land Surface Temperature/Emissivity products here, as Kampala is located in a very cloudy region due to the bi-annual overpassing of the intertropical convergence zone and lake-induced convection [77–79]. The accuracy of the MODIS Collection 6 (C6) Land Surface Temperature product (MOD11) has been assessed over a widely distributed set of locations and time periods via several ground-truth and validation efforts [80]. MODIS Collection based approach has been found to be acceptable even in locations with frequent cloud cover within global SUHI studies [56, 81]. Generally, the MODIS LST accuracy is better than 1°C in the range from −10 to 50°C [82]. The fine space-based climatic information provided by MODIS LST has been validated for detailed spatial assessment of climate variability in Africa, such as in Egypt [83] and other locations in north Africa [80].

To detect the annual daytime LST change in the Kampala urban cluster, we used GEE to generate LST from the MODIS average 8-Day (MOD11A2.006) within 100 km^2^ from city centre in 2003 and 2017.

To quantify SUHI effects in Kampala over time in relation to urbanisation, we used the Global Surface UHI Explorer within GEE to retrieve annual daytime SUHI intensity. The SUHI dataset was created based on the simplified urban-extent (SUE) algorithm [63], by using the concept of an urban cluster and rural areas as the basis for the calculation of SUHI intensity. An urban cluster is more appropriate than using administrative boundaries, as the latter can frequently change over time. Equally, administrative boundaries are sometimes not comparable across cities and often do not include the full extent of a city [56, 57]. Our chosen approach allows us to focus on the locations where the expansion and SUHI is occurring. In the SUE algorithm, SUHI is calculated from the difference between the mean of the land surface temperature (LST) over the urban land use pixels, or the non-urban, non-water land use pixels. These two subsets of pixels are based on the urban extent dataset MODIS land cover classification (MCD12Q1.051) and the European Space Agency Climate Change Initiative (ESA CCI) land cover data.

To detect the variability of SUHI and extent of impervious surfaces within the Kampala urban cluster, change detection analysis was used to designate differences between images of the same area at different times. The change detection approach was applied intervals 2003 and 2017, which provides “from–to” change information and the type of change that has occurred can be easily calculated and mapped [84]. The change areas are illustrated using mosaic plot methods to provide a visual representation of SUHI intensity losses and gains and urban built-up expansion.

There are some limitations in our research that need to be acknowledged. First, we only discuss changes in SUHI, whether positive or negative. However, what might bring about the changes cannot be discerned in detail from our approach. Second, our results illustrate what might be expected from the expansion of urban areas, while there is relatively little detail on the actual urban surface cover or its characteristics through the methods used. Further considerations would include topography, morphology and biodiversity of the study cities [85, 86]. These features could be explained more by using alternative approaches, such as a local climate zone maps [87–89], which is our next step in this research.

## Results

### Historical patterns of urbanisation

**Fig 2:**
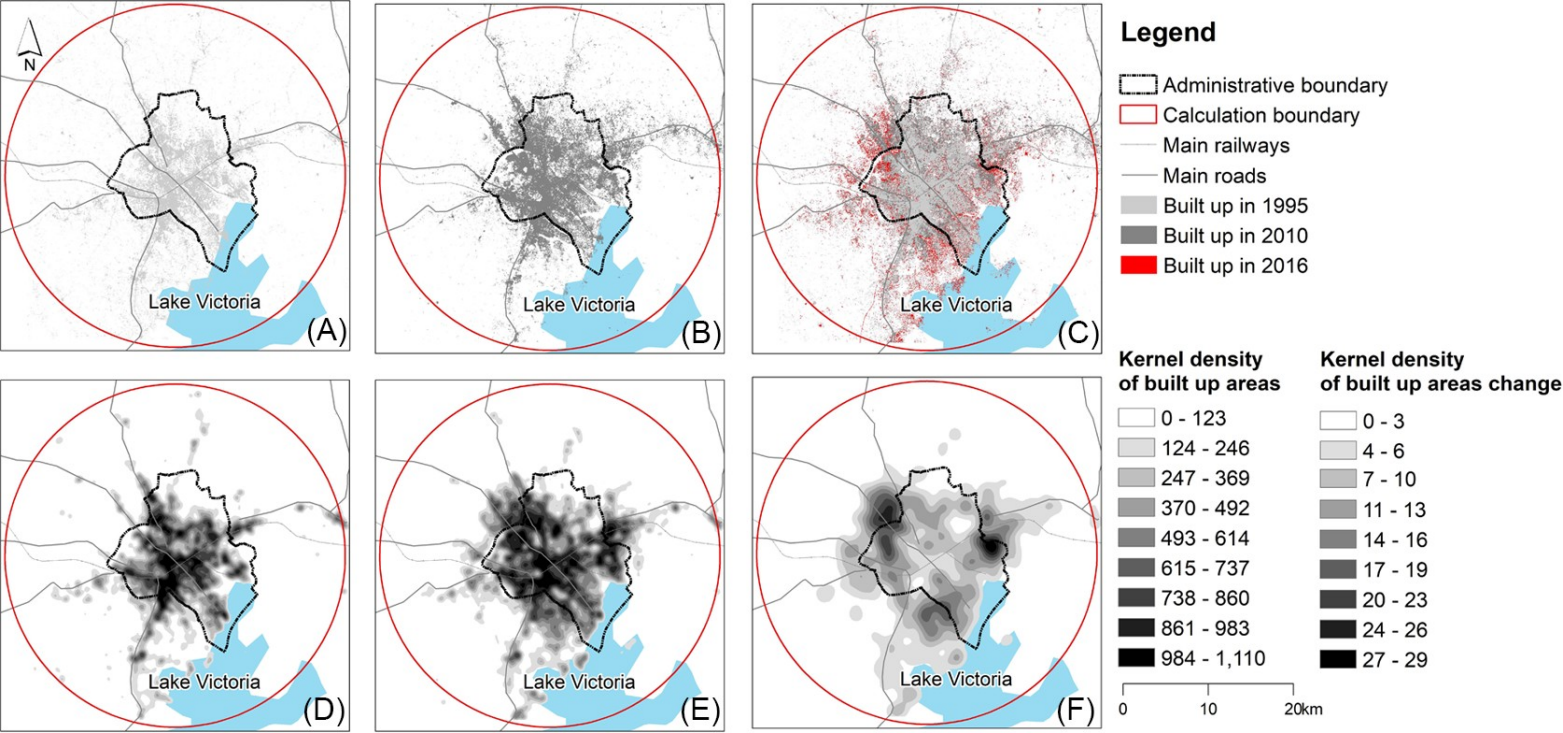
The built-up distribution of Kampala in 1995 (A), 2010 (B) and 2016 (C); the built-up kernel density of Kampala in 1995 (D), 2016 (E) and the new built-up area in 2016 compared to 1995 (F) significantly or show strong evidence of infilling urbanisation developments. The classification method is equal interval, with nine classes and a white to black color scheme to point towards high (black) and low (white) point pattern densities. The highest point pattern densities represent the hotspots of city growth from 1995 to 2016, defined as compact growth within the city.

Between 1995 and 2016, Kampala underwent extensive changes to its urban built-up area (Fig 2). Urban land cover extended from the centre of the city to adjoining non-built up areas in all directions, but in particular to the southwest, east, northwest and north along major transport corridors. The urban built-up area of Kampala was 25,389 ha in 2016, representing an average annual rate of increase of 6.9% since 2010. The urban built-up area in 2010 was 15,952 ha, increasing at an average annual rate of 1.8% since 1995, when its urban built-up area was 12,133 ha. In total, 13,256 ha of non-built up land has been converted to urban land use. Different parts of the city experienced different patterns of development. Fig 2D–2F shows KD outputs for built-up areas in 1995, 2016 and the new built-up areas in 2016 compared to 1995, and their default value, respectively. The central region consolidated over time and the eastern regions underwent relatively compact growth, while the southwest and western regions showed fragmentation followed by infill and expansion at the edge of the city. The northern and the peri-urban regions were the most fragmented, with scattered areas of urban land cover, indicative of leapfrog development.

### Changes in land surface temperatures and SUHI effect

**Fig 3:**
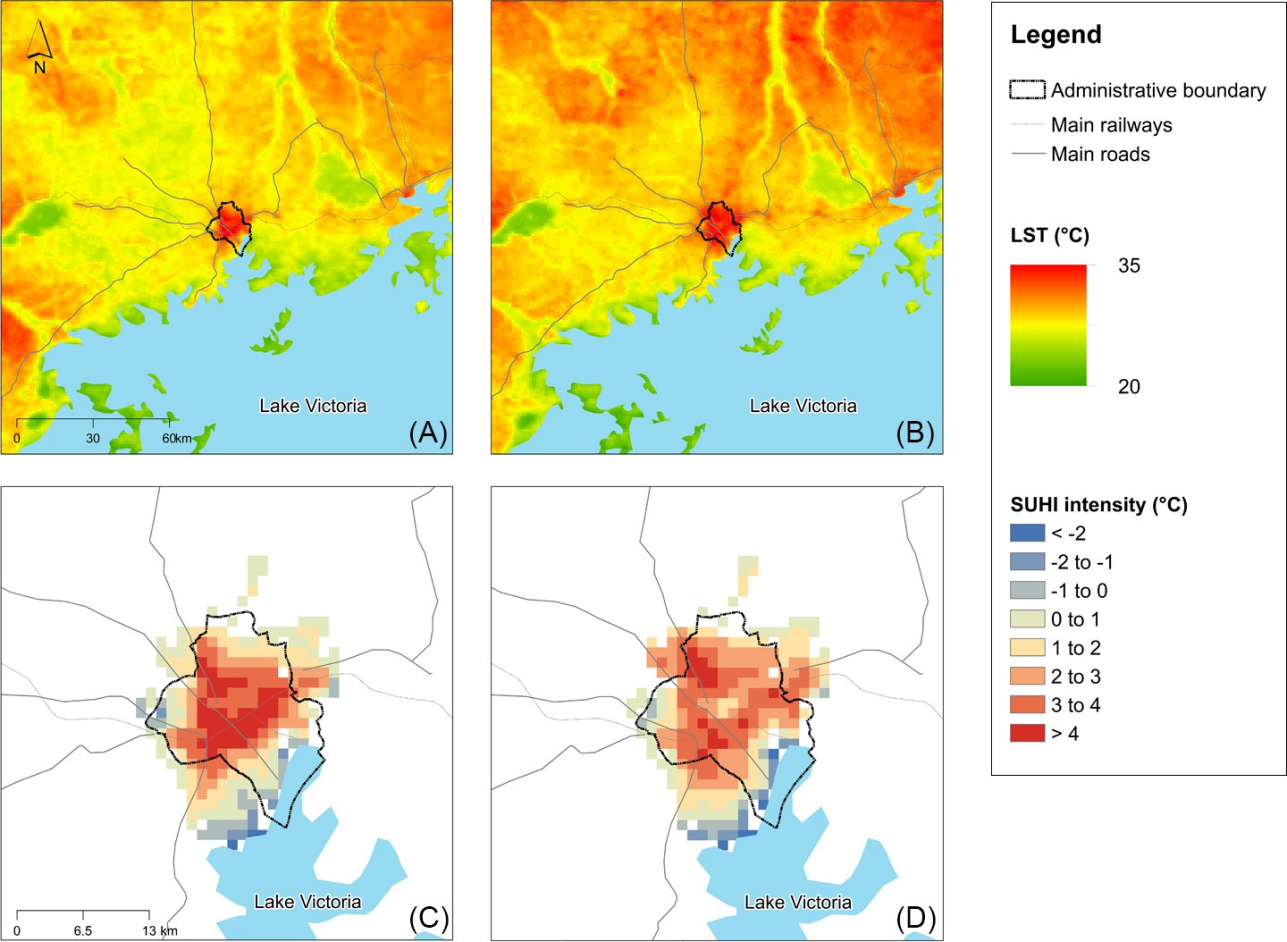
Average annual daytime temperature in 2003 (A) and 2017 (B) for Kampala and the surrounding region. Annual daytime SUHI intensity of Kampala in 2003 (C) and 2017 (D).

In 2003, the annual LST within the Kampala urban cluster was 26.5°C. This increased to 26.8°C in 2017 (Fig 3). In both years, the built-up areas of Kampala were hotter than the surroundings, indicating a SUHI effect (Fig 3C and 3D). In 2003, the annual daytime SUHI was 2.2°C, and 1.9°C in 2017. The SUHI intensity area expanded from 22,910 ha in 2003 to 27,900 ha in 2017.

The areas affected by SUHI expanded in two different ways that directly linked to patterns of urbanisation, namely (i) infilling areas that were already experiencing a SUHI, and (ii) extending SUHI into areas of the region that were previously unaffected. By 2017, SUHI intensification was spreading from the centre of the Kampala urban cluster towards the north, northwest and east. These areas were relatively unaffected by SUHI effects in 2003. At the same time, the intensity of the SUHI effect in the centre of the urban cluster dropped.

**Fig 4:**
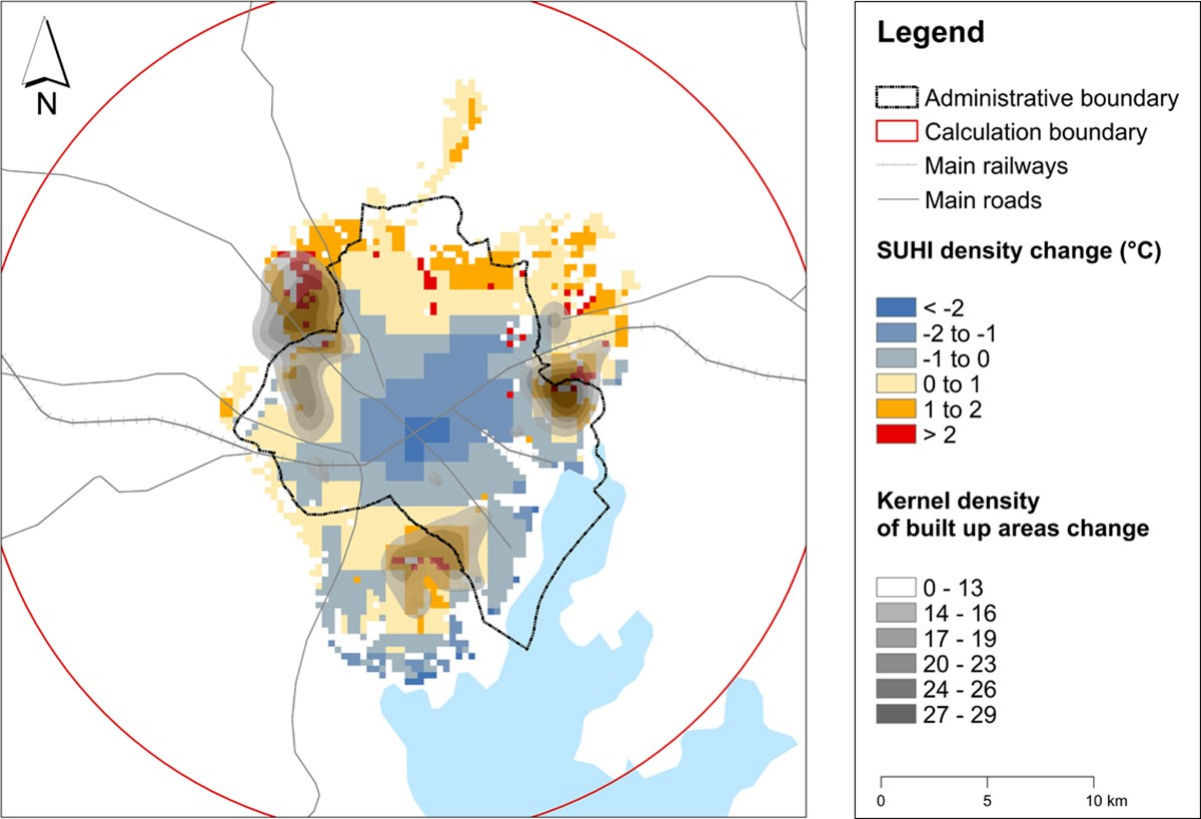
Daytime SUHI value distributions for Kampala urban cluster in 2003 and 2017. The width of each curve corresponds with the approximate frequency of data points. There is a small box plot in the middle of each density curve, with the rectangle showing the ends of the first and the third quartiles and central line the median. The n value under the x-axis is the number of SUHI pixels.

The violin plots (Fig 4) show the different distribution of daytime SUHI in 2003 and 2017 in the Kampala urban cluster [90]. The widest width of the curve which indicated the peak frequency distribution located in the third quartile in 2003 moved to the first quartile with a longer tail in 2017. The changes in the violin plots could be explained by the difference spatial distribution of the SUHI between the two years. The frequency of SUHI value presents an overall rise (the peak of violin plot width), with more below negative SUHI outliers present (longer tail of the violin plot in 2017).

In 2003, the highest intensity of SUHI effect (5.9°C) was in the centre of the urban cluster, this dropped to 3.9°C in 2017. The mean annual LST also drop from 34.7°C to 34.0°C. The highest intensity of SUHI effect in 2017 was 4.3°C, and this was located in northwest of the urban cluster where in 2003 the SUHI was 4.4°C. And the mean annual LST increased from 32.9°C to 34.7°C. In other words, the intensity of SUHI experienced in areas that were already affected in 2003 became less intense, but new areas, subject to even more intense SUHI effects emerged. Further, the southeast and east of the urban centre became cooler relative to the surrounding rural areas–and urban cooling effect. The largest cooling effect also shifted from the very south of the urban cluster (-2.0°C in 2003, -2.8°C in 2017) in 2003 to the south and south east (non-urban areas in 2003, -3.0°C in 2017) by 2017. Annual mean LST of these two locations both increased in 2017, the former increased from 26.5°C to 27.7°C and the latter rose from 27.4°C to 28.2°C. The reduced SUHI and the cooling effect is likely due to the proximity of Lake Victoria.

**Fig 5:**
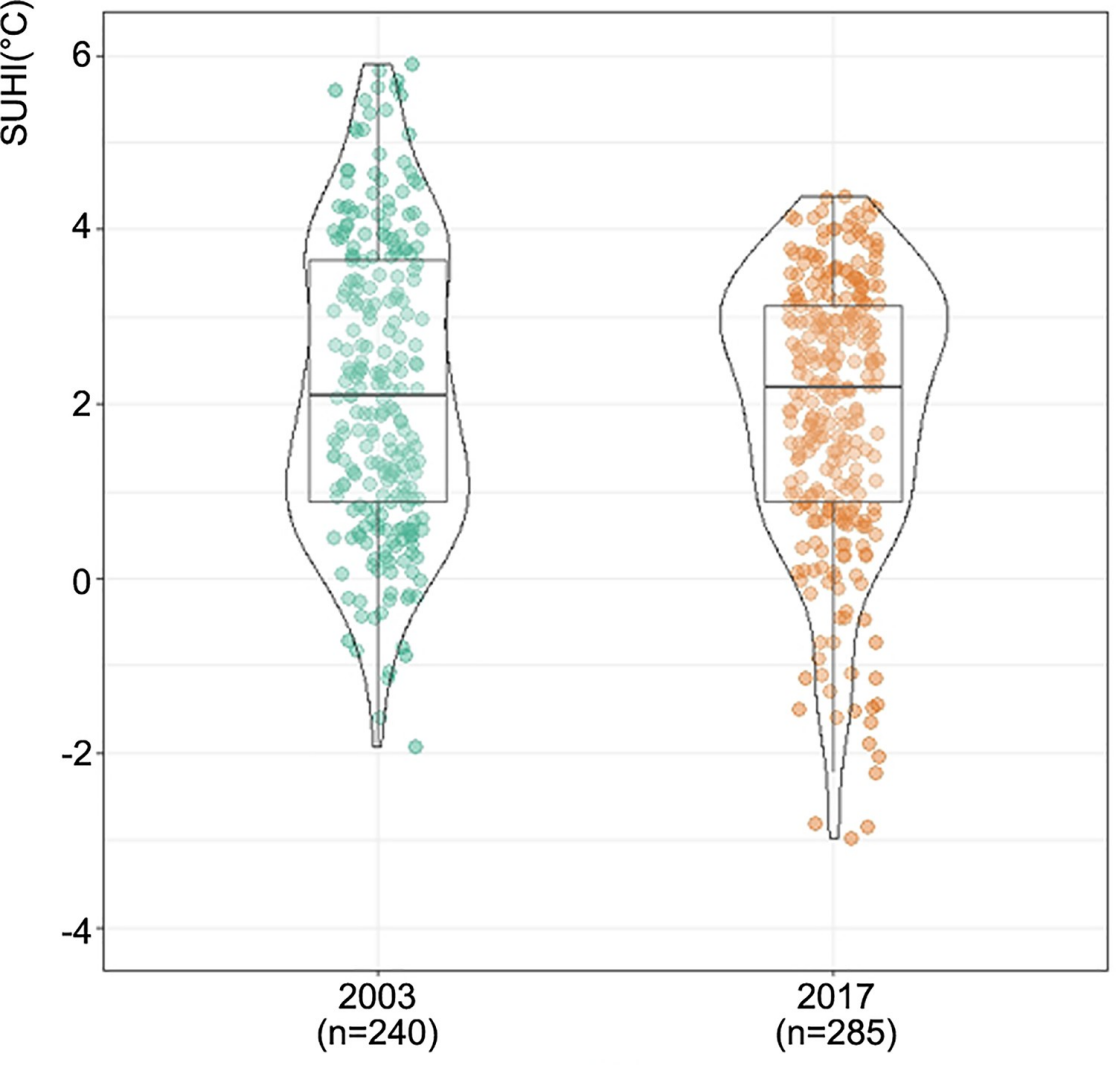
The SUHI intensity change (2003–2017) compared with the built-up change (1995–2017).

Changes in SUHI intensity were spatially coincident with alterations in the distribution of built-up land cover within the Kampala urban cluster (Fig 5). Although patterns of change were similar, they were not totally synchronized. The areas exhibiting the greatest SUHI intensity change were towards the north and northeast of the urban cluster, but these parts of the city did not expand.

## Discussion

Kampala is situated on the northern shores of Lake Victoria, and has experienced exponential urban population growth since 1970 [37–39], largely influenced by rural-urban migration [91]. Fast urban population growth requires the construction of more infrastructure and the conversion of greater areas of land to coverage by built structures, such as buildings and sealed surfaces. We found that the spatial patterns of urbanisation in Kampala showed urban sprawl along radial corridors, such as major traffic lines from the city centre, as well as a more general spread around the edges of the existing urban areas [39, 54]. This is a very common phenomenon, both historically in high-income countries in the past [92] and across many cities in Africa at present [93–95]. The SUHI affected areas of the Kampala urban cluster also expanded concomitant with the urbanisation process. In particular, the relentless urban built-up land expansion at the expense of bluespace or green space (such as wetland, cropland and forest) has caused the SUHI effect to increase in certain areas of the city and its fringe [93, 96].

Such rapid urbanisation could increase the vulnerability of informal communities to natural hazards as green and blue spaces are converted to built uses. Over 60% of the population live in informal settlements in Kampala [47], and many of these unplanned developments occur in low-lying areas which are vulnerable to natural hazards [45]. Green and blue spaces can provide important climate change mitigation services, such as carbon sequestration and shade through presence of trees in greenspaces, so their loss has important implications.

As in many other cities, rapid urbanisation in Kampala is characterised by poor and vulnerable groups moving into large informal settlements [45, 97]. In these parts of the city, SUHI was exacerbated, perhaps caused by the presence of informal settlements in peri-urban areas that create densely built-up sites. Houses in informal settlements are often poorly constructed, which means that many are unlikely to withstand climate impacts and lack adequate air circulation or insulation, which is particularly threatening for vulnerable groups during heat waves [9, 98]. Even these SUHI intensity changes do not provide direct evidence of the unprecedented challenge of human health, wellbeing and economic development [99]. Such transformations could enhance the vulnerabilities of informal communities in this climate and environmentally sensitive area. Indeed, the 50-year flood zone map developed by World Bank Group [100], suggests the high risk flooding areas in Kampala are largely located in middle and low employment density areas, which overlap with emerging SUHI intensity and urban built-up sprawl areas identified in the present study.

In contrast, in the south east of the city, near Lake Victoria, SUHI is negative, and the intensity of SUHI has decreased over time. This is likely to be due to the urban breeze effect, which will have been strengthened as the urban area changed. Former studies have highlighted the importance of lake breezes resulting from the thermal contrast between land and water surfaces [79]. Urbanisation can impact the intensity of the main wind flows, either increasing or decreasing its intensity [79, 101]. Also, changes in city roughness could impact lake breezes, and modulate wind velocities [101].

The interaction between the global phenomenon of climate change and the local phenomenon of the SUHI effect [102], driven by an accelerating urbanisation path is creating a potentially dangerous heat stress risk that is expected to increase. However, in most East African cities, the problem of SUHI has received less attention, as planners instead put emphasis on water management and flood control in city development plans [103–105]. Since there is a close connection between these two phenomena of flooding and SUHI [25], extra warming caused by SUHI may lead to greater upward motion and turbulence of the air, which in turn induces precipitation [25, 27]. Urban surface roughness, and the presence of aerosols from air pollution can also induce precipitation [25, 27]. There has therefore been a growing recognition that both flooding and SUHI need to be tackled together [53], and efforts to mitigate the SUHI effect are gradually becoming an increasing priority in city planning [43].

For the Kampala urban cluster, one study has suggested that sustainable urban development should use restrictive urban planning measures to stop wetland encroachment, developing satellite towns to secondary urban hubs, and building a more structured metropolitan area [39]. In the meantime, contributions of greenspace and bluespace to urban cooling should be considered in Kampala’s urban future development. Greenspace and bluespace (e.g., green roofs, green alleys and streets, urban forests, parks, gardens, wetlands) are often highlighted as providing significant ecosystem services: improving residents’ health and wellbeing, providing food, lowering wind speeds, reducing storm-water run-off, modulating ambient temperatures, reducing energy use and sequestering carbon [106, 107]. Bluespace and green space can mitigate the SUHI effect, providing cooling benefits, important for reducing energy consumption and improving human health and well-being [108, 109]. Research has suggested that tree dominated greenspace offers greater heat stress relief while improperly designed bluespace may exacerbate heat stress [109]. To address the SUHI effect in Kampala, a collaborative effort between scientists and urban planners to devise contextually relevant strategies is needed [110].

Related impacts can be anticipated from urban development combined with potentially more frequent occurrences of extreme climatic events such as heat stress and flooding [25]. Kampala is located in a tropical rainforest climate zone (Köppen climate classification) with two dry seasons per year (December to February and June to July). SUHI exacerbation in this area during the dry season has been detected by previous research [111]. Urban residents in the most built-up parts of the urban areas are the most vulnerable to heat stress, as well as heat-related health complications [49]. Accompanied with rising poverty, and vulnerability of the urban poor to these impacts, these combined issues are bringing about greater awareness for the need to sustainably mitigate and adapt to climate change risks [98, 110]. Increased temperature combined with flooding has introduced health challenge and caused a surge in disease outbreaks of malaria, dysentery and cholera since 1997’s El Nino oscillations [112, 113]. To cope with these, basic urban infrastructure such as drainage, supply of sanitation and water are required and need to adequately maintained [113]. To enhance adaptive capacity, more equitable and adequate access to basic urban infrastructure should be a priority for poorer communities [114]. In addition innovative community-based adaptations coupling climate change impacts and poverty reduction have proved effective [115], including domestic energy briquettes from waste, productive greening and urban agriculture, as well as household-level rainwater harvesting and nutrient recycling from waste [113, 116].

The growth of cities will inevitably continue and successfully managing city growth will be critical to economic development and social stability [104]. Climate change and its impacts cannot be tackled by a single solution that is universally applicable. Since 2015, Uganda government has been adopting explicit National Urban Policies (NUPs) for coordinated policy responses to enhance sustainable urban growth in line with Sustainable Development Goal (SDG #11) and the New Urban Agenda (NUA). For instance, Uganda’s NUP highlights that: “*The SDGs provide an opportunity for Uganda to bring all stakeholders together to decide and embark on new paths to improve the lives of people in urban areas*, *and to make cities and human settlements inclusive*, *safe*, *resilient and sustainable by creating mechanisms to ensure good urban governance*” [117]. Even though governments are becoming more sensitive to the threats and opportunities posed by the process of rapid urbanisation [118], identifying appropriate implementation pathways to achieve the policy aspirations is still a challenge, as NUPs remains high-level policy expressions, with little impact in practice [119]. NUPs are conducive to but not sufficient to achieve sustainable cities [120], and require concrete action plans and tools to enforce practical actions on the ground [118].

The urban design approach will need to vary according to the humidity and the amount of ventilation needed [87, 121], as climate change and urbanisation not only affect the land and lake breezes over the Lake Victoria region but also alter the urban humidity [79]. In particular, the enhanced surface temperatures associated with UHI can actually result in shorter vegetation growing seasons; a contrast with results reported for temperate cities [122]. SUHI mitigation strategies using bluespace and green space could potentially realise synergies for SUHI mitigation and urban flooding [104], as well as support adaptation to climate change [123]. The possibility of harnessing both mitigation and adaptation benefits from bluespace and greenspace opens up a ‘window of opportunity’ to achieve climate compatible development, by simultaneously realising double- or triple-wins [124, 125].

## Conclusions

This study tracked the dynamics of annual SUHI intensity in the Kampala urban cluster, Uganda, and their response to urban built-up area changes over the period 2003–2017. We extracted the urban built-up area using GEE for the study area between 1995 and 2017, then explored the SUHI growth patterns and their responses to the urbanisation processes. We used Global Surface SUHI Explorer for retrieval of annual daytime SUHI intensity to reduce uncertainties in SUHI estimation and to investigate the long-term variability of the SUHI [56]. Our analysis allowed us to reach the following conclusions:

1. The expansion of SUHI effect areas and urban built-up areas in Kampala urban cluster is substantial in the study period. The main increment of SUHI effect areas in 2017 appeared in the peri-urban parts of the study area, accompanied with SUHI effect mitigation in the urban cluster centre. In particular, SUHI in Kampala occurred as a spatial process of diffusion and coalescence, originally being located around the urban cluster centre, and subsequently stretching across previously unaffected areas, and detracting from the centre.
2. The SUHI effect areas of Kampala urban cluster show expansion concomitant with the urbanisation process. Relentless expansion of urban built-up land at the erosion of bluespace or greenspace (e.g. wetland, cropland and forest) has caused the SUHI effect impacted areas to be dramatically expended. The fast urbanisation could enhance the vulnerabilities of informal communities with the loss of bluespace and greenspace in rural and peri-urban areas, particularly where strong institutions for risk management are lacking.
3. Kampala is one of the most populated cities in GLR region, and is growing in a fragmented-complex way [126], with urban expansion partly accompanied with densification [127]. Fast urbanisation and climate change accompany complex and changing land-lake-atmosphere interactions, requiring city planners and policy makers to carefully determine the direction of their urban growth strategies and consider the cooling effect provided by greenspace and bluespace. Efforts should be more targeted to ensure that they optimise the health and wellbeing of vulnerable inhabitants by maximising ecosystem services, as well as utilising collaborative approaches involving different groups of stakeholders [128].
